# Chronic Deafness Degrades Temporal Acuity in the Electrically Stimulated Auditory Pathway

**DOI:** 10.1007/s10162-018-0679-3

**Published:** 2018-07-02

**Authors:** John C. Middlebrooks

**Affiliations:** 1 Department of Otolaryngology, University of California at Irvine, Irvine, CA 92697-5310 USA; 2 Center for Hearing Research, University of California at Irvine, Irvine, CA USA; 3 Department of Neurobiology and Behavior, University of California at Irvine, Irvine, CA USA; 4 Department of Cognitive Sciences, University of California at Irvine, Irvine, CA USA; 5 Department of Biomedical Engineering, University of California at Irvine, Irvine, CA USA; 6 Department of Language Science, University of California at Irvine, Irvine, CA USA

**Keywords:** cochlear implant, intraneural, cat, auditory nerve, degeneration, phase locking

## Abstract

Electrical stimulation of the auditory nerve with a penetrating intraneural (IN) electrode in acutely deafened cats produces much more restricted spread of excitation than is obtained in that preparation with a conventional cochlear implant (CI) as reported by Middlebrooks and Snyder (J Assoc Res Otolaryngol 8:258–279, 2007). That suggests that a future auditory prosthesis employing IN stimulation might offer human patients greater frequency selectivity than is available with a present-day CI. Nevertheless, it is a concern that the electrical field produced by an IN electrode might be too restricted to produce adequate stimulation of the partially depopulated auditory nerve of a deaf patient. We evaluated this by testing responses to IN and CI stimulation in adult-deafened cats. Activation of the auditory pathway was monitored by recording from the central nucleus of the inferior colliculus (ICC). Cats deaf for 153–277 days exhibited a ~ 30 % loss of auditory nerve fibers compared to cats deaf for < 18 h. Contrary to our concern, measures of thresholds and dynamic ranges showed no significant deafness-related impairment of excitation by IN or CN stimulation. Surprisingly, however, temporal acuity decreased dramatically in these adult-deafened cats, as demonstrated by a marked decrease in the maximum rate of electrical cochlear stimulation to which ICC neurons synchronized to IN or CI stimulation. For instance, half of ICC neurons synchronized to IN stimulation up to 203 pulses per second (pps) in acute deafness, whereas that number dropped to 79 pps for chronic deafness. Such a loss of temporal acuity might contribute to the poor sensitivity to temporal fine structure that has been reported in human CI users. Seemingly, the degraded temporal acuity that we observed in cats was even worse than the fine-structure sensitivity of human CI users, suggesting that most patients experience some improvement of temporal acuity resulting from restoration of patterned auditory nerve stimulation by a CI.

## Introduction

The standard of care for bringing hearing sensitivity to severely-to-profoundly deaf people is a cochlear implant (CI) consisting of an array of electrodes inserted into the scala tympani of the cochlea. Most present-day CI users can expect to achieve usable speech reception in quiet surroundings. Success with CIs, however, varies widely among users (Gifford et al. 2008; Holden et al. 2013), and nearly all CI users exhibit impaired sensitivity to temporal fine structure (Zeng 2002; van Hoesel 2007; Kong et al. 2009; Laback et al. 2015), leading to impaired temporal pitch sensitivity and impaired spatial hearing.

Several factors have been proposed to account for the variability in performance among post-lingually deaf CI users (reviewed by Moberly et al. 2016). One widely held view is that the success with a CI declines with increasing duration of profound deafness prior to implantation (Gantz et al. 1993; Blamey et al. 1996; UK Cochlear Implant Study Group 2004; Holden et al. 2013). Nevertheless, several recent studies have failed to show an appreciable effect of duration of deafness (Medina et al. 2017), particularly after eliminating a confound of age at testing (Beyea et al. 2016). Others have shown a marked influence of duration of deafness on CI performance only for durations of deafness > 30 years (UK Cochlear Implant Study Group 2004; Moon et al. 2014).

One reason that long duration of deafness might contribute to impaired CI performance is that loss of cochlear hair cells could lead to secondary loss of spiral ganglion cells and auditory nerve fibers. Cats and guinea pigs can show nearly 100 % loss of Type I spiral ganglion cells and auditory nerve fibers after more than a year following ototoxic or acoustic insult (Ylikoski 1974; Spoendlin 1975; Webster and Webster 1981; Leake and Hradek 1988). In humans, however, the post-deafening loss of spiral ganglion cells is not nearly so rapid (Otte et al. 1978; Nadol et al. 1989). Nadol et al. (1989) studied the post mortem temporal bones of deaf patients in some cases decades after the onset of profound deafness. Spiral ganglion cells counts in that study were reduced by only ~ 25 to 75 % of normal, depending primarily on etiology of deafness. Moreover, studies of the temporal bones of CI users have shown little or no relationship between success in CI use and post mortem spiral ganglion cell counts (Blamey 1997; Khan et al. 2005a, b; Fayad and Linthicum 2006); one study showed that, in each of six individual bilaterally implanted listeners, better word recognition was obtained with the ear having more ganglion cells, but those within-listener differences were small and ganglion cell count was not predictive of word recognition across listeners (Seyyedi et al. 2014).

We previously have tested in an acutely deafened animal model a novel mode of auditory prosthesis consisting of a multi-electrode array penetrating into the auditory nerve (Middlebrooks and Snyder 2007, 2008, 2010). By every metric that we evaluated in anesthetized cats, that intraneural (IN) stimulation was superior to stimulation obtained with a conventional CI. In particular, the tonotopic spread of excitation by IN stimulation was substantially more restricted. In a future clinical prosthetic employing IN stimulation, that restricted excitation might offer a user improved frequency selectivity. It is a concern, however, that the restricted electrical field of an IN electrode might not excite enough fibers in the partially depopulated auditory nerve of a deaf patient to reach threshold or to provide adequate growth of loudness. To address that concern, we tested responses to IN and CI stimulation in cats that had been deafened as adults and left unstimulated for around 6 months (referred to as “chronically deaf”). Activation of the auditory pathway was monitored by recording from the central nucleus of the inferior colliculus (ICC), and auditory nerve fiber survival was evaluated histologically.

The chronically deaf animals lost ~ 30 % of their auditory nerve fibers compared to adult animals deafened and studied within 18 h. Despite that fiber loss, they showed essentially no change in thresholds or dynamic range for IN or CI stimulation compared to acutely deafened animals. An unanticipated finding was that transmission of temporal fine structure from the electrically stimulated cochlea to the ICC was markedly degraded. It remains to be tested whether such loss of temporal acuity is irreversible, or whether acuity might be restored to some degree by the restoration of patterned auditory nerve activity brought about by electrical stimulation.

## Methods

### Overview

Cats were deafened and then studied in a terminal physiological experiment either on the day of deafening (acute) or 153–277 days after deafening (chronic); the acute deaf data were taken from previous studies (Middlebrooks and Snyder 2007, 2010), whereas the chronic deaf data were new to this study. The terminal experiment consisted of recordings from the central nucleus of the inferior colliculus (ICC) during electrical stimulation of the contralateral ear. The electrical stimulation used an animal version of a conventional CI (a banded electrode array) inserted in the scala tympani and a thin-film IN electrode array inserted into the auditory nerve. Physiological measures of interest were thresholds for activation of recording sites in the ICC, the dynamic range over which ICC activity was tonotopically restricted, and the maximum electrical pulse rate at which ICC neurons phase locked significantly to cochlear electrical pulse trains. Following the end of physiological data collection in each animal, the auditory nerves were evaluated histologically.

### Subjects

The 12 cats that were chronically deafened and evaluated in the present study were purchased from a research breeding colony at the University of California at Davis. The deafening, terminal physiological experiments, and histology using those animals were conducted at the University of California at Irvine, with approval by the University of California at Irvine Committee on Use and Care of Animals. Histological data came from 13 ears of 8 of the chronically deaf cats. Control histological data came from six ears of three additional cats from the Davis colony that were acutely deafened in Irvine. Control physiological data from 15 acutely deafened cats are taken from previously presented studies conducted at the University of Michigan :10 cats from a study of spread of excitation and dynamic ranges (Middlebrooks and Snyder 2007) and 5 cats from a study of temporal acuity (Middlebrooks and Snyder 2010). Both of those studies were conducted with approval from the University of Michigan Committee on Use and Care of Animals. Those animals were purchased from Harlan Sprague Dawley (Indianapolis, IN) and Liberty Research, Inc. (Waverly, NY).

### Deafening

Acute and chronic deafening procedures targeted cochlear hair cells. Any subsequent loss of spiral ganglion cells or nerve fibers was assumed to be largely secondary to hair cell loss.

For the *chronic* deafening, 12 normal-hearing adult animals were anesthetized with an intramuscular injection of ketamine (33 mg/kg). Baseline acoustic auditory brainstem responses (ABRs) were recorded to a click intensity series, and normal thresholds (0–20 dB SPL) were confirmed for both ears. Animals were then deafened bilaterally using the ototoxic protocol described by Moore et al. (2002). Briefly, kanamycin (300 mg/kg dissolved in sterile saline) was injected subcutaneously. After a delay of ~ 30 min, a solution of ethacrynic acid dissolved in saline (1 mg/ml) was administered via intravenous infusion. The infusion was continued until no click-evoked ABR response was obtained at equipment maximum (~ 90 dB SPL); total ethacrynic acid doses ranged from 55 to 100 mg, median = 80 mg. ABR responses were monitored for 4 h to ensure that hearing thresholds did not immediately recover. After the initial deafening procedure, animals were re-tested periodically to determine whether any hearing recovery had occurred. In 3 of the 12 cases, partial recoveries of ABR click thresholds (to ≤ 60 dB SPL) were observed 76–78 days after the original deafening procedure, prompting a second course of kanamycin and ethacrynic acid. The 12 chronically deaf animals were group housed for 153 to 276 days from the initial deafening to the terminal experiment.

As described previously (Middlebrooks and Snyder 2007, 2008, 2010), *acute* deafening was performed on the day of the terminal experiment while the animal was already under barbiturate anesthesia. The right cochlea was deafened by ablation of the ossicular chain. Recordings were made from the right ICC in response to acoustic stimulation of the intact left ear. Then, the round window of the left cochlea was opened, perilymph of the first cochlear turn was removed with a cotton wick, and neomycin sulfate (10 % in distilled water) was instilled. This eliminated ICC responses to acoustic stimulation within ~ 10 min. All of the physiological data in the acutely deafened animals were collected within 18 h of deafening.

### Terminal Physiological Experiments

Each of the 12 chronically deaf animals was studied in a terminal physiological experiment that was largely identical to the previously reported studies that yielded the control data from 15 acutely deafened animals (Middlebrooks and Snyder 2007, 2010). The only substantive difference was that responses to acoustical stimulation could not be used in the chronically deaf animals to identify the tonotopic position of the ICC recording array, so an alternative procedure was used as described below.

Stimulus generation and data acquisition used System 3 equipment from Tucker-Davis Technologies (TDT; Alachua, FL) with custom MATLAB software (The Mathworks, Natick, MA). Electrical stimuli were delivered using a custom optically isolated constant-current 16-channel stimulator.

Two types of cochlear stimulating arrays were evaluated in the terminal experiments: intrascalar CIs and IN arrays. Intrascalar arrays (Cochlear Americas, Centennial, CO) were identical to the distal eight bands of a conventional human banded CI. Intraneural (IN) arrays consisted of 16-site, single-shank silicon-substrate arrays (NeuroNexus Technologies, Ann Arbor, MI, USA). The stimulation sites on the IN arrays were activated iridium, 703 μm^2^ in area, spaced at 100-μm intervals along the shank. The CI and IN stimulation both used a monopolar configuration, with the active electrode consisting of a selected intrascalar or intraneural electrode and the return path consisting of a platinum-iridium wire in a neck muscle. All 15 of the acutely deaf animals were tested with both CI and IN electrodes. Of the 12 chronically deaf animals, 6 were tested with both CI and IN electrodes and 6 were tested only with IN electrodes. In addition to the CI and IN devices, a ball electrode placed at the cochlear apex was used to aid in identifying the tonotopic map in the ICC in the chronically deaf animals (as described below); the ball electrode was a ~ 0.25-mm-diameter ball flamed on the end of a silver wire.

Each animal was anesthetized with ketamine followed by intravenous sodium pentobarbital, supplemented as needed to maintain an areflexive level of anesthesia. The right inferior colliculus was exposed by opening the skull and aspirating overlying brain tissue. An intrascalar CI was implanted in the left scala tympani. The CI array was inserted through the round window and along the scala tympani with the most basal band (designated electrode #1) lying at the junction of the basal turn with the hook region of the cochlea. The most apical stimulating electrodes (designated electrodes #7 and #8) lay at approximately the middle of the second turn. The apex of the left cochlea was exposed and a ball electrode was placed on the osseous spiral lamina.

The ICC recording array consisted of a single silicon-substrate shank with 32 recording sites spaced at 100-μm intervals (NeuroNexus Technologies, Ann Arbor, MI). The array was inserted into the inferior colliculus, oriented in the coronal plane and ~ 40 ° from the mid-sagittal plane, from dorsolateral to ventromedial. That trajectory was roughly parallel to the tonotopic axis of the ICC. Our previous studies have used responses to tonal stimulation prior to deafening to identify the tonotopic positions of recording sites. Instead, in the present study with chronically deaf animals, we positioned the recording array such that: (1) short-latency responses to electrical stimulation could be recorded on all 32 recording sites; (2) stimulation of basal intrascalar sites activated deep ICC sites, in the presumed high-frequency representation; and (3) stimulation of the apical ball activated superficial ICC sites in the presumed low-frequency representation. The success of that procedure for positioning the ICC recording array was confirmed by subsequent findings that the topography of ICC responses to IN stimulation showed the characteristic spiral pattern that we have observed in previous studies (Middlebrooks and Snyder 2007, 2008; described in the “Results” section). When a satisfactory position was achieved, the ICC recording array was fixed in place.

Responses to stimulation of the intrascalar CI electrodes were evaluated, and then the CI was explanted and the IN array was inserted as follows: (1) the margins of the round window were enlarged, (2) a hole was made in the osseous spiral lamina using the beveled tip of a 30-ga hypodermic needle, and (3) the IN array was inserted through the hole into the underlying auditory nerve using a micro-positioner. In a previous report (Middlebrooks and Snyder 2008), we referred to this as the “transcochlear” (as opposed to the “posterior fossa”) approach. The IN array was oriented across the trunk of the auditory nerve, approximately perpendicular to the long axis of nerve fibers. The depth of the IN array was adjusted, or the array was withdrawn and re-inserted if necessary, so that all IN stimulation sites elicited activity in the ICC, as much as possible following the previously described spiral topography.

Measures of thresholds and dynamic ranges for CI and IN stimulation used single biphasic electrical pulses, initially cathodic, 41 μs per phase with no inter-phase gap. Stimulus sets varying in CI or in IN stimulus site and current level (typically in 1-dB steps) were presented pseudo-randomly, repeated at onset-to-onset intervals of 300 ms until each combination of site and level had been tested 20 times.

Measures of temporal acuity used 300-ms trains of 41-μs-per-phase biphasic pulses varying in pulse rate in steps of 40 pulses per second (pps) from 40 to 600 pps. For CI stimulation, typically the most apical CI site was tested. The apical CI electrode was selected because stimulation thresholds typically were lowest on that electrode and because we wished to provide the greatest spread of activation to apical cochlear fibers that was possible with our CI electrodes. For IN stimulation, 2 to 6 IN stimulation sites (median = 3) were selected to sample ICC responses to stimulation of apical, basal, and sometimes middle-turn fibers; for that reason, individual ICC recording sites were represented 1 to 6 times in each data set and, in some conditions, the total sample size was greater than 384 (i.e., greater than 32 sites times 12 cats). Current levels of the pulse trains were 4 and 8 dB above the estimated detection threshold of units at the most sensitive ICC recording site; in some cases, additional levels at 2-dB increments were tested. The trains were repeated at onset-to-onset intervals of 600 ms until each combination of stimulus site, pulse rate, and level, varied pseudo-randomly, had been tested 20 times.

The neuronal waveforms recorded from the 32 ICC sites were digitized simultaneously, displayed on-line, and stored on computer disk. A simple on-line peak picker was used to detect action potentials for the purpose of monitoring ICC responses and setting stimulus parameter ranges. All reported data, however, are based on off-line spike sorting. Electrical artifact from the cochlear stimulus was eliminated on-line by a sample-and-hold function that was programmed into the recording path (Middlebrooks and Snyder 2008). Artifact rejection entailed holding the recorded signal constant for a 143-μs period synchronized with each 82-μs biphasic electrical pulse. That procedure resulted in loss of ~ 0.6 to 8.6 % of the waveform around each pulse for the 40-to-600-pps pulse trains that were tested; responses to single pulses occurred well after recovery from the electrical artifact. In a few instances in which adequate artifact rejection could not be attained for pulse trains, and during test cases in which the artifact rejection was disabled, artifact propagated to the ICC with group delay of ~ 2 ms. Such short-delay activity was distinct from neural activity (with group delay > 4 ms) and was eliminated from further analysis.

### Analysis of Physiological Data

Neural action potentials were discriminated off-line using a spike-sorting procedure described previously (Middlebrooks 2008). Among the 12 animals new to the present study, 1 to 9 (mean 5.1) of the 32 ICC recording sites in each animal yielded recordings of well-isolated single neurons. Additional spike activity, consisting of unresolved spikes from two or more neurons, could be recorded on nearly all other recording sites of all probes. All recorded extracellular spike activity will be referred to here as “unit activity,” except in cases in which we specifically refer to “well-isolated single units”. The number of ICC recording sites and the number of units included in any specific analysis varied according to the number of sites activated by the specific stimulus and the number of stimulus sites that were tested.

The pulse train stimuli elicited stimulus-synchronized non-spike near-field potentials that could have contaminated spike sorting. In the present and in the previous study (Middlebrooks and Snyder 2010), that potential contamination was mitigated by processing the neural waveforms as follows prior to spike sorting: (1) the waveforms at each recording site were averaged across all 20 repetitions of each unique set of stimulus parameters to form an estimate of the field potential and (2) that estimate was subtracted from individual waveforms. The action potential spikes were brief in duration and not precisely entrained across trials. For that reason, each individual spike contributed little to the estimate of the slow-wave potential and remained after subtraction of the estimate.

**Fig. 1:**
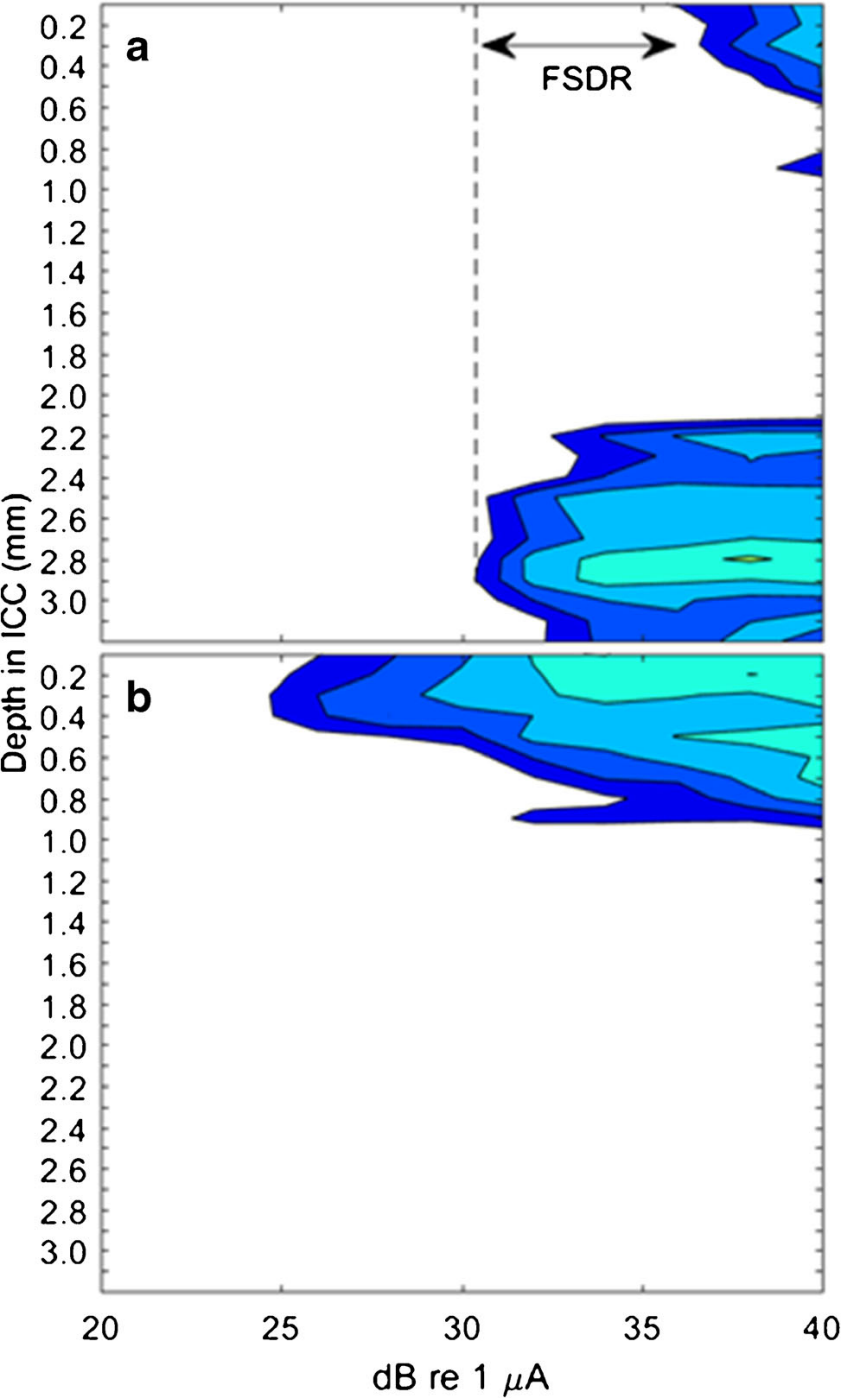
Spatial tuning curves (STCs) for two intraneural stimulation sites recorded in chronically deaf cat IN1204. Each of these sets of contours plots the sensitivity index, *d’*, in steps of 1 *d’* unit. The vertical axis shows depth along the recording array in the central nucleus of the inferior colliculus (ICC), and the horizontal axis shows the stimulus current. Stimuli were single biphasic pulses, initially cathodic, 41 μs/phase. The stimulus site yielding the STC in **a** was relatively superficial in the nerve. The lowest stimulus levels excited only basal fibers (indicated by activity deep in the ICC). At higher levels, excitation spread to apical fibers. FSDR indicates the frequency-specific dynamic range, the range of currents over which activity was restricted to the lowest-threshold tonotopic region. The stimulus site in **b** was deeper in the nerve and activated only apical fibers over the range of currents that was tested

Thresholds for detection of single electrical pulses were computed based on trial-by-trial spike counts using a procedure derived from signal detection theory (Green and Swets 1966; Macmillan and Creelman 2005; Middlebrooks and Snyder 2007). Background spike counts were measured in the interval 18 to 3 ms preceding each stimulus pulse, and stimulus-driven spike counts were measured in the interval 3 to 18 ms following the pulse. For each stimulus level, we formed an empirical receiver operating characteristic (ROC) curve based on the trial-by-trial distributions of background and driven spike counts. The area under the ROC curve gave the probability of correct detection, which was expressed as a *z*-score and was multiplied by √2 to obtain the sensitivity index, *d’*. Contours of *d’* plotted versus stimulus level and depth along the ICC tonotopic axis were referred to as spatial tuning curves (STCs; Fig. 1). The lowest stimulus value at which *d’* was ≥ 1 was taken as the threshold for a particular stimulus condition. The range of stimulus levels over which the STC was tonotopically restricted with no spread to noncontiguous regions was taken as the frequency-specific dynamic range (FSDR). The STC in Fig. 1a is from an instance in which fibers from the basal turn were excited at lowest current levels, resulting in low-threshold activation in the high-frequency end of the tonotopic axis deep in the ICC. In this case, excitation spread at higher current levels to apical fibers. In this example, the threshold was 28.6 dB re 1 μA and the FSDR was 7.8 dB. The STC in Fig. 1b, from the same cat, shows selective activation of apical fibers, with threshold of 24.8 dB re 1 μA. In this instance, there was no spread of activation to noncontiguous regions within the range of currents that were tested, so the FSDR was recorded as > 15.2 dB.

As stated above, current levels of the electrical pulse trains were 4 and 8 dB above the threshold of the most sensitive unit across all 32 recording sites. Analysis of the responses of each ICC unit to cochlear electrical pulse trains was conducted at the lowest tested level at which *d’* for electrical stimulation of that unit was ≥ 1. Given the considerable variation of thresholds across the 32 ICC recording sites, stimulus levels at which pulse train responses were analyzed varied across sites from 0 to 4 dB above threshold. That relatively low level was chosen because many units could not be tested at higher levels, either because of the larger electrical artifact at higher levels or because the stimulator could not generate sufficiently high currents to activate some high-threshold ICC units at higher supra-threshold levels. The strength of phase locking of ICC neurons to electrical pulse trains was estimated by computing vector strength (Goldberg and Brown 1969). The statistical significance of vector strengths was evaluated using the Rayleigh test of circular uniformity (Mardia 1972) at the level of *p* < 0.001. The limiting rate for each unit was the highest pulse rate at which the vector strength was statistically significant (i.e., greater than the Rayleigh criterion). Units in the ICC responded to the onset of pulse trains with a temporally compact burst of spikes, regardless of the pulse rate. That initial response would have given an erroneous impression of precise phase locking. For that reason, vector strength was computed based on spikes occurring during the interval 50–300 ms after the onset of the pulse train.

Reported first-spike latencies are from the responses to the first pulses of electrical pulse trains. We selected every trial in which there were one or more spikes > 3 ms after the first electrical pulse. The first-spike latency was given by the median latency of the first spike across those selected trials. Latencies < 3 ms were regarded as too short to have resulted from cochlear stimulation and, thus, were interpreted as spontaneous.

### Histology

At the end of each terminal experiment, the animal was euthanized with an intravenous injection of an approved euthanasia solution and then perfused transcardially with warm Ringer’s solution followed by cold (~ 4 °C) histological fixative (0.5 % glutaraldehyde, 2 % paraformaldehyde, 4 % sucrose in 0.1 M phosphate buffer titrated to pH 7.4). After vascular perfusion, the temporal bones were harvested and trimmed to the petrous temporal bone, while leaving the auditory nerve intact with the lateral portion of the cochlear nucleus attached to the nerve. The cochlear round windows were opened by careful dissection, and the cochleas were perfused by intrascalar lavage with the same histological fixative. The cochleas were then stored for at least 24 h in the vascular fixative at 4 °C. After fixation, the cochleas were rinsed in several changes of phosphate buffer at 4 °C for several days. Then, while still in cold buffer, the bone surrounding the internal auditory canal was removed using a diamond burr. This exposed the central portions of the auditory, vestibular, and facial nerves. The cochleas were post-fixed by immersion in 1 % osmium tetroxide in 0.1 M phosphate buffer for 24–48 h; osmium stains myelin dark. Then, the cochleas were rinsed in phosphate buffer at room temperature, and decalcified by placing them on a rotator in 20-ml vials filled with 3 changes of 2 % ethylenediaminetetraacetic acid (EDTA) at room temperature for at least 24 h. After decalcification, the cochleas were dehydrated in graded concentrations of ethanol (50–100 %), placed in 2 changes of propylene oxide, and embedded in a mixture of Epoxy and Araldite resins (Ted Pella, Tustin, CA).

**Fig. 2:**
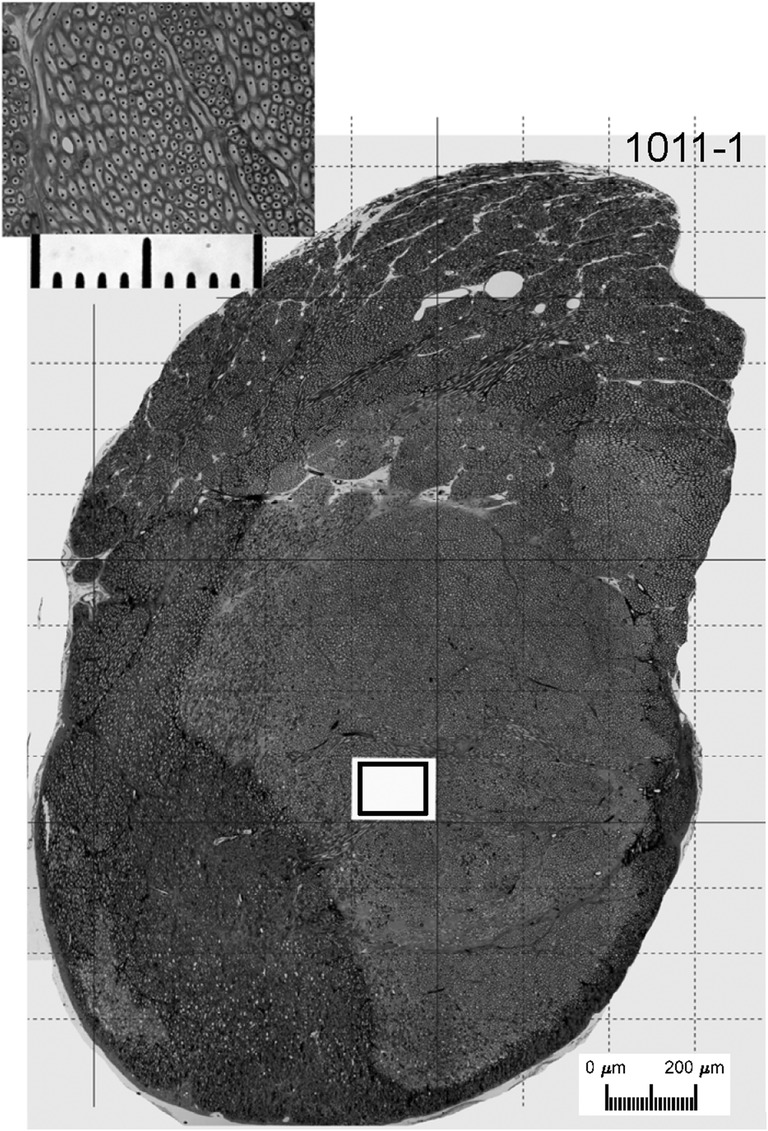
Photomontage of section through an auditory nerve from normal-hearing Cat1011. The large image is a photomontage of images taken at × 200 magnification. Based on the photomontage, the estimated area of this nerve was 2.59 mm^2^. The fibers in the upper one third of this very peripheral section are still separated into the fascicles, which is a characteristic of the fibers as they travel from Rosenthal’s canal and coalesce into the nerve within the internal auditory canal. The grid lines indicate the potential sites from which non-overlapping high-magnification (× 1000) samples were selected randomly. The outlined box inside the white rectangle shows the size of such a sample. The inset shows a single high-magnification sample. The overall length of the corresponding scale bar is 100 μm. The black dots in the high-magnification image were added as an aid in counting each myelinated axon profile

Glass knives and a Sorvall MT2-B microtome were used to cut 2-μm serial cross sections of the auditory nerve beginning at the internal auditory meatus and proceeding laterally (i.e., toward the cochlea). Sample sections were placed on glass slides and cover slipped with immersion oil. Successive sample sections were collected until a level beyond the lateral extent of the cochlear nucleus was reached but before the nerve began to break up into fascicles which run out to the spiral ganglion. At this level, overlapping digital images (taken at × 200 magnification) of one entire section through the auditory nerve were captured on a Zeiss photomicroscope. A photomontage of each whole nerve cross-section, with a calibration scale, was constructed from these images using Photoshop CS5™. The area of the nerve cross section was estimated by tracing its perimeter in ImageJ (NIH) and using its measuring software. A grid was then superimposed upon each montage. A randomly distributed sample of non-overlapping grid rectangles (5 to 17, median 12) was then chosen with the provision that the entire area within each rectangle lay entirely within the perimeter of the nerve. A high resolution × 1000 image within each chosen rectangle was taken, representing an area of 0.0148 mm^2^. The number of myelinated nerve fibers within each high-resolution image was counted by superimposing a black dot in the middle of each myelinated fiber (see inset in Fig. 2) and then counting the dots using the ImageJ particle analysis tool. The number of fibers in each nerve was estimated from the mean number of fibers in the 0.0148-mm^2^ samples and the overall area of the nerve cross section.

## Results

### Loss of Auditory Nerve Fibers during Chronic Deafness

We evaluated possible effects of chronic deafness on the auditory nerve by estimating the number of surviving myelinated nerve fibers in control and chronically deaf cats. The control data came from six auditory nerves in three acutely deaf cats. The chronically deaf data came from 13 nerves in 8 of the cats that were studied in terminal experiments, 8 nerves from the ear that was implanted with a CI and an IN array during the terminal experiment and 5 from the non-implanted ear. There was no significant difference in nerve counts between implanted and non-implanted ears (Wilcoxon signed rank test, *p* = 0.44, d.f. = 4).

Figure 2 illustrates a 2-μm section through an auditory nerve from one control cat. The low-magnification image is a montage of images taken using a × 20 objective, and the higher-magnification image in the inset was taken using a × 100 oil-immersion objective. In the right side of the low-magnification montage, one can see a somewhat distinct fascicle of fibers, which we take to be high characteristic-frequency axons from the basal cochlear turn. Additional, less distinct, fascicular organization can be seen throughout the montage. Myelinated nerve profiles were counted in the high-magnification digital images (like that shown in the inset) by marking each profile with a dot and then counting the dots using the ImageJ particle analysis tool; the dots can be seen in the inset. The cross-sectional area of the nerve, 2.59 mm^**2**^ in this example, was computed from a tracing of the low-magnification montage. That area along with the mean of fibers per high-magnification sample, 383.6 in this example, permitted an estimate of the total number of myelinated fibers in the nerve, 67281 in this example. Across the 6 nerves that we examined from three acutely deafened cats, nerve areas ranged from 1.89 to 2.59 mm^2^ (mean = 2.28 mm^2^), average fiber counts per high-magnification sample, averaged across each nerve, ranged from 341.1 to 480.5 fibers, and coefficients of variation for the counts in the 8-to-15 samples of each nerve (100 × standard deviation/mean) ranged from 8.8 to 20.3 (mean = 13.4). The estimated fiber counts in each control nerve ranged from 54,423 to 76,649 fibers (mean = 65,712).

**Fig. 3:**
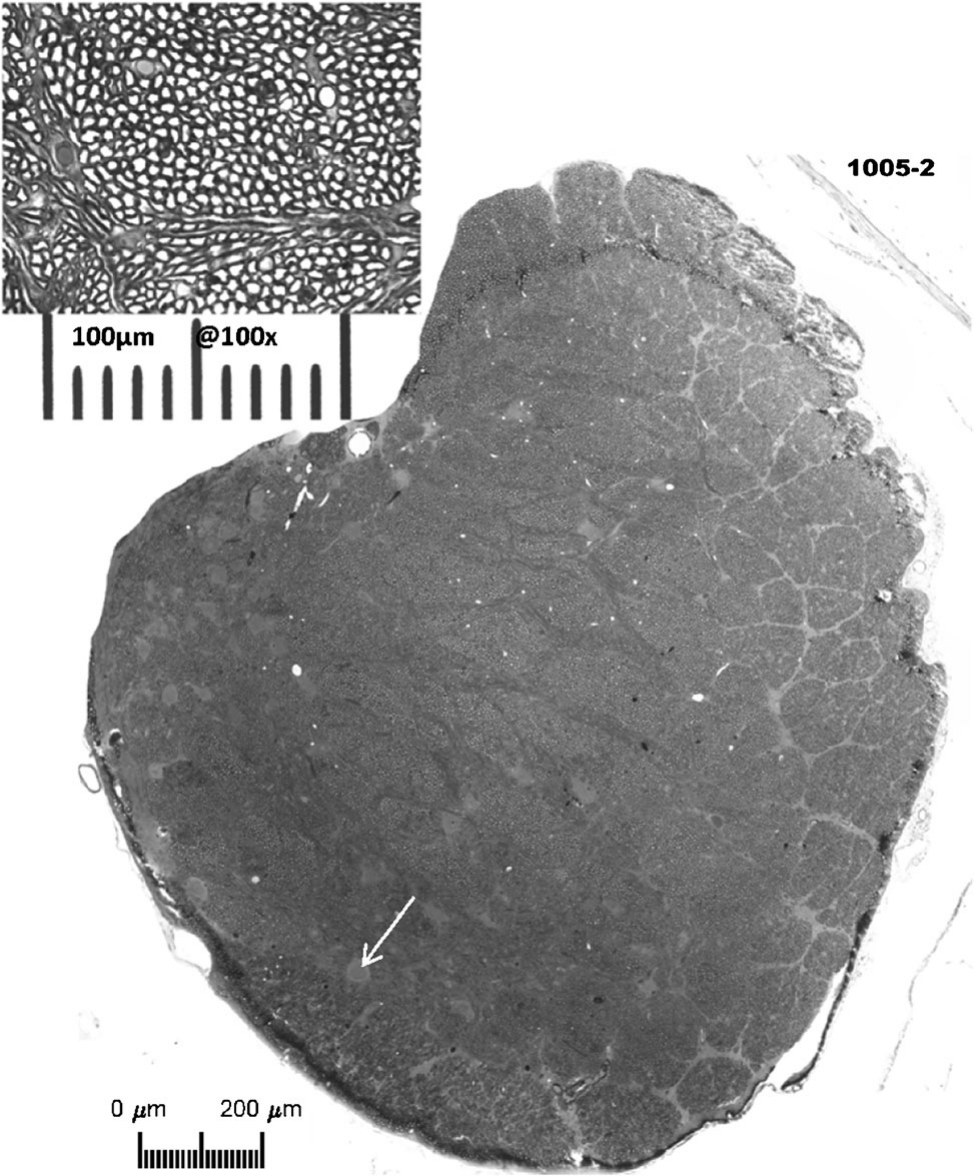
Photomontage of the left auditory nerve from chronically deafened Cat1005. The estimated area was 1.47 mm^**2**^. The total number of fibers in this section was estimated to be 45,259. Note the nerve cells (e.g., white arrow) around the left periphery of the montage of this very peripheral section

Figure 3 shows a section through the left auditory nerve of a chronically deaf cat. This section was taken at a level that is very peripheral (close to the cochlea) along its course through the internal auditory canal from the cochlea to the cochlear nucleus. The fascicular organization is more pronounced in this example than in the example in Fig. 2, particularly along the right side. One can see in this section a large number of cell bodies embedded within the nerve (e.g., white arrow). Such neuron cell bodies were commonly seen along the length of the nerve in both control and chronically deaf animals. These neurons were most frequently observed in the nerve near the internal auditory meatus, but we observed them as far peripherally as the fundus of the modiolus. On the basis of their appearance in our osmium-stained material and their location, we take these neurons to be homologous to the cochlear root neurons that have been characterized in small rodents (Osen et al. 1991). The cross-sectional area of this nerve was 1.47 mm^**2**^, the mean number of nerve fibers per high-magnification sample was 495.4, and the estimated total number of nerve fibers was 49,289. Across the 13 nerves that were examined from chronically deaf cats, overall cross-sectional area of the nerve ranged from 1.38 to 2.02 mm^2^ (mean = 1.72 mm^2^), fiber counts per high-magnification sample averaged 282.8 to 519.7 fibers (mean = 395.3 fibers), and coefficients of variation for the counts in the 5-to-17 samples of each nerve ranged from 9.5 to 34 (mean = 17.8). The estimated fiber counts in each control nerve ranged from 29,671 to 58,867 fibers (mean = 45,827).

**Fig. 4:**
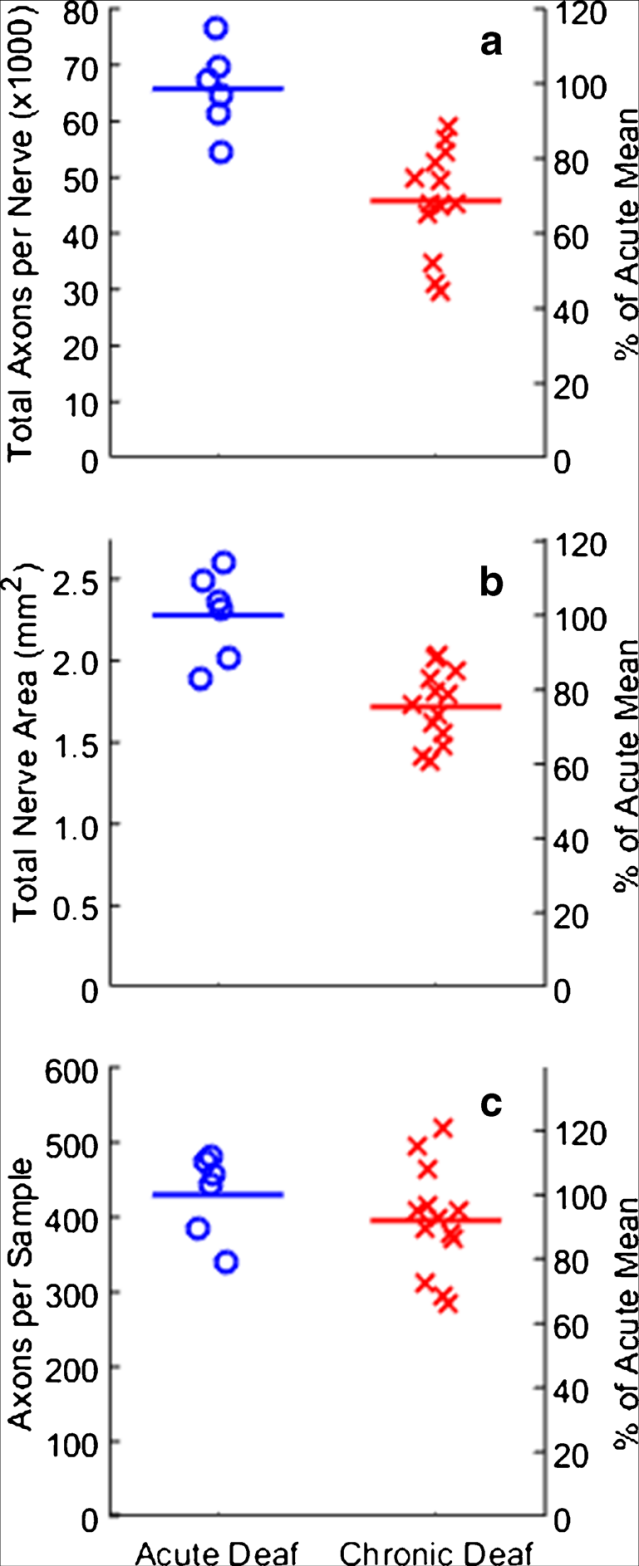
Nerve fiber populations in 6 auditory nerves taken from 3 normal-hearing, acutely deafened cats (blue o’s), and in 13 nerves taken from 8 chronically deaf cats (red x’s). **a** Total fiber count per nerve. The left axis gives raw fiber counts. **b** Total area of each nerve. **c** Mean number of fibers (averaged within each nerve) within each 0.0148-mm^2^ sample. In each panel, the right axis expresses values as percentages of the mean in the acute deaf condition

The chronically deaf nerves showed a substantial loss of fibers compared to the acutely deafened nerves. Figure 4a shows the estimated fiber counts expressed as counts (left axis) and as percentages of the mean of control counts (right axis). The fiber counts in the chronically deaf nerves were reduced by a mean of 30.3 % (45,827 versus 65,712, two-sample *t* test: *p* = 0.00029, *t =* 4.5, *d.f.* = 17). The fiber loss was particularly evident as reduction of the overall cross-sections of chronically deaf nerves (Fig. 4b; 1.72 versus 2.28 mm^2^, two-sample *t* test, *p* = 0.00017, *t =* 4.8, *d.f.* = 17). The mean number of fibers in the high-magnification samples was somewhat lower in the chronically deaf nerves (Fig. 4c; 395.3 versus 430.0), but the difference was not significant (two-sample *t* test, *p* = 0.31, *t =* 1.0, *d.f.* = 17).

### Excitability and Topography after Chronic Deafness

The basic responses of ICC neurons to single electrical pulses from the intrascalar CI and from the IN array were largely similar to those that we have reported previously for acutely deaf cats (Middlebrooks and Snyder 2007, 2008).The responses to CI stimulation were tonotopically broad, showing widely overlapping shifts in the pattern of ICC activation as the intrascalar stimulating electrode was shifted from most basal to most apical. The number of useable stimulation sites was somewhat reduced in the chronically deaf animals because of the reduced diameter of the auditory nerves, but the IN stimulating arrays generally could be positioned such that all 16 electrodes lay within the auditory nerve, even in the narrowed nerves of the chronically deaf animals. The responses to IN stimulation showed the characteristic spiral topography from stimulus site to ICC tonotopic locus, similar to what we have encountered in acutely deafened animals (Middlebrooks and Snyder 2007, 2008). That is, proceeding from superficial to deep sites in the auditory nerve: (1) the most superficial sites on the IN stimulation array, presumably situated among fibers from the basal cochlear turn, activated deep, high-frequency sites in the ICC with low thresholds; (2) in all 12 chronically deaf animals there was a hiatus along the IN stimulating array, a few 100 μm in length, in which thresholds were elevated and, in 9 of those cases, individual IN sites elicited bimodal patterns in the ICC involving the highest and lowest ends of the recording array (as in Fig. 1a); (3) deep to the hiatus, sites in the nerve presumably from the apex activated superficial, low-frequency, sites in the ICC; and (4) in 7 animals, successively deeper IN sites activated ICC tonotopic loci progressing from the most superficial (low-frequency) tonotopic sites to deeper sites corresponding to the middle cochlear turn and intermediate frequencies. Based on this characteristic deep to superficial to intermediate topography of responses in the ICC, we assigned IN stimulation sites to basal, apical, and (in seven cases) middle cochlear turns. That we did not consistently locate the middle-turn fibers has been our experience even in acutely deafened animals.

**Fig. 5:**
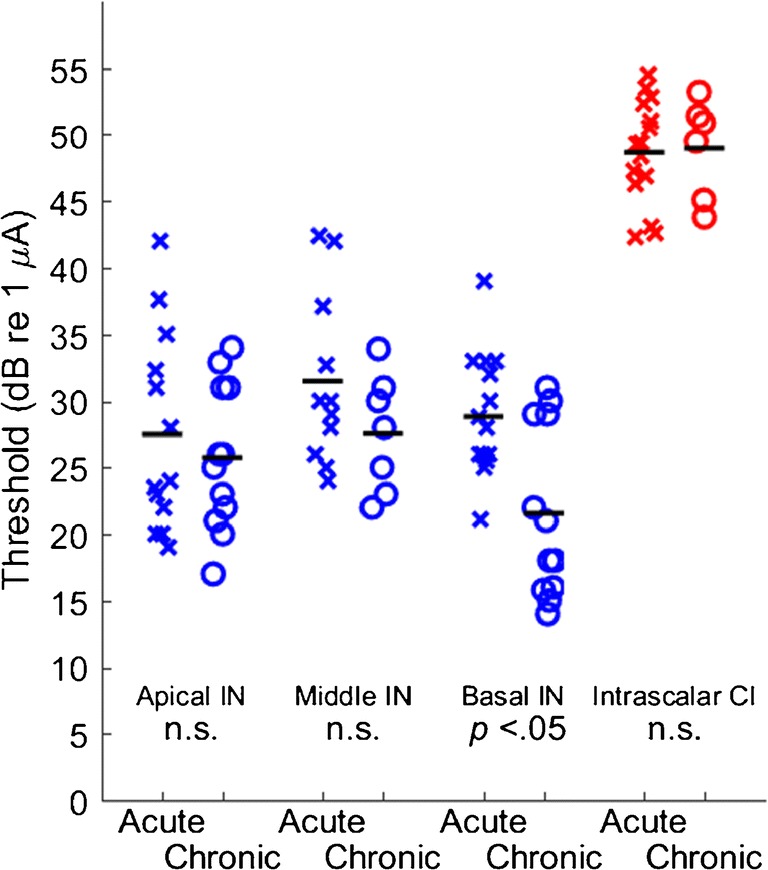
Thresholds for activation of ICC neurons by electrical cochlear stimulation. X’s and o’s indicate acute and chronic deafness conditions, respectively. Stimuli were single biphasic pulses, initially cathodic, 41 μs/phase, presented in a monopolar configuration. Data for intraneural (IN) stimulation, shown in blue, represent the lowest-threshold stimulus site in each animal eliciting apical-, basal-, or (when present) middle-turn patterns of activation. Data for intrascalar cochlear implant (CI) stimulation, shown in red, represent the lowest-threshold CI stimulus site in each animal, typically the most apical site. Symbols are jittered in the horizontal dimension to improve visibility. The horizontal lines indicate mean values for each condition. Only the basal IN thresholds were significantly different between acute and chronic deafness conditions

We tested the hypothesis that the excitability of the auditory nerve might be compromised by a loss of fibers; that was particularly a concern in the case of IN stimulation in which the spread of the electrical field could be quite restricted. The results in the 12 chronically deaf cats did not support that hypothesis, neither for intrascalar CI nor for IN stimulation. We evaluated excitability by measuring the thresholds for activation of ICC neurons by single 41-μs/phase biphasic electrical pulses through a single CI or IN site, as discussed in the “Methods” section and shown in Fig. 1. Figure 5 plots the thresholds from the acutely and chronically deaf animals. The intrascalar CI data are from the CI electrode yielding the lowest threshold in each animal, typically one of the two most apical electrodes. The IN data are given for the sites yielding the lowest thresholds for stimulation of apical-, basal-, and (when available) middle-turn fibers.

Across both acutely and chronically deaf animals, thresholds were substantially lower for IN than for CI stimulation. Thresholds for IN stimulation, across all cochlear turns and both conditions of deafness, averaged 27.9 dB re 1 μA, whereas CI thresholds, across both conditions of deafness, averaged 48.8 dB re 1 μA (ANOVA: *p* < 10e^−6^, *F* = 210, d.f. = 1,89). Because of the > 20-dB difference in thresholds between IN and CI stimulation, we examined effects of deafness condition separately for CI and IN.

The CI stimulation showed no significant difference in threshold between acute and chronic deafness (ANOVA: *p* = 0.87, *F* = 0.026, d.f. = 1,19). An ANOVA was performed for IN stimulation, with factors of condition of deafness and cochlear turn. That analysis showed a small, unexpected, *reduction* in threshold in the chronically deaf condition (*p* = 0.0046, *F* = 8.6, d.f. = 1,64); no significant difference in thresholds among apical, middle, and basal turns (*p* = 0.071, *F* = 2.8, d.f. = 2,64); and no significant two-way interaction (*p* = 0.25, *F* = 1.4, d.f. = 2,64). Post hoc analysis of effects of condition of deafness on threshold for each of the three cochlear turns showed a significant effect only for the basal turn (*p* < 0.05 after Bonferroni correction).

**Fig. 6:**
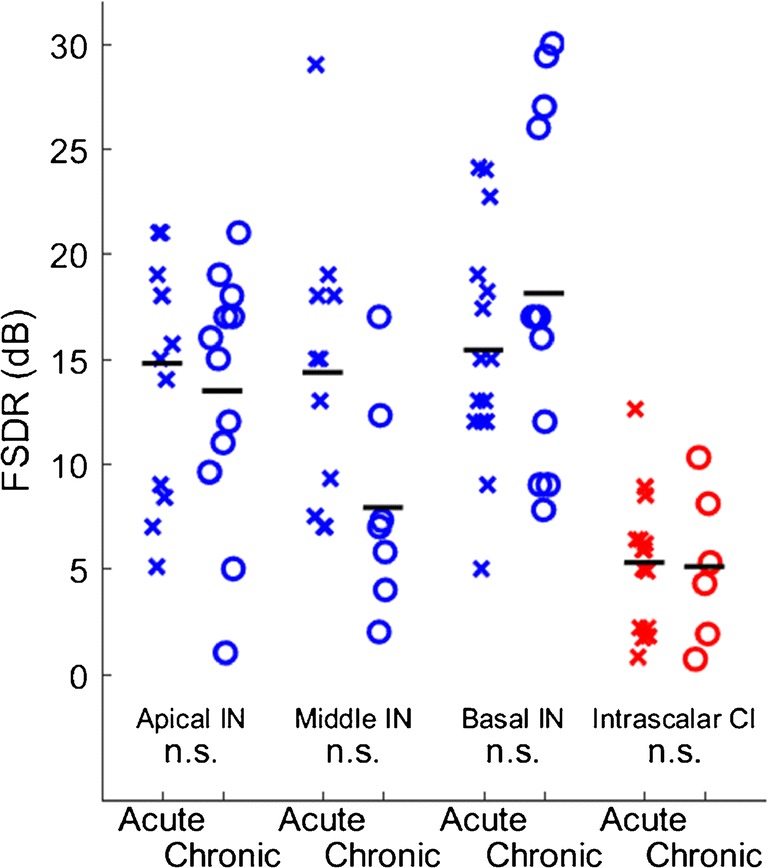
Frequency-specific dynamic ranges (FSDR) for activation of ICC neurons. The definition of FSDR is illustrated in Fig. 1. Other format details are as in Fig. 5. None of the conditions showed a significant difference between acute and chronic conditions of deafness

We computed the frequency-specific dynamic range (FSDR) as a measure of the range of stimulus levels over which the spread of tonotopic activation was contained without spreading to noncontiguous tonotopic loci; an example is given in Fig. 1. Figure 6 shows the distributions of FSDR across both conditions of deafness, three cochlear turns for IN stimulation, and CI stimulation. The FSDRs for IN stimulation, across both conditions of deafness and three cochlear turns, averaged significantly broader than did FSDRs for CI stimulation, averaged across both conditions of deafness (*p* < 1e^−6^, *F* = 38, d.f. = 1,89). The FSDRs for CI stimulation showed no significant difference between acute and chronic deafness (*p* = 0.91, *F* = 0.014, d.f. = 1,19). A two-way ANOVA of FSDRs for IN stimulation showed no significant effect of condition of deafness (*p =* 0.28, *F* = 1.2, d.f. = 1,64), a weak but significant effect of cochlear turn (*p* = 0.019, *F* = 4.2, d.f. = 2,64), and no significant two-way interaction (*p =* 0.072, *F* = 2.7, d.f. = 2,64). A post hoc analysis showed no significant effect (*p* > 0.05) of the condition of deafness on FSDR for any of the individual cochlear turns.

### Temporal Acuity Degraded by Chronic Deafness

**Fig. 7:**
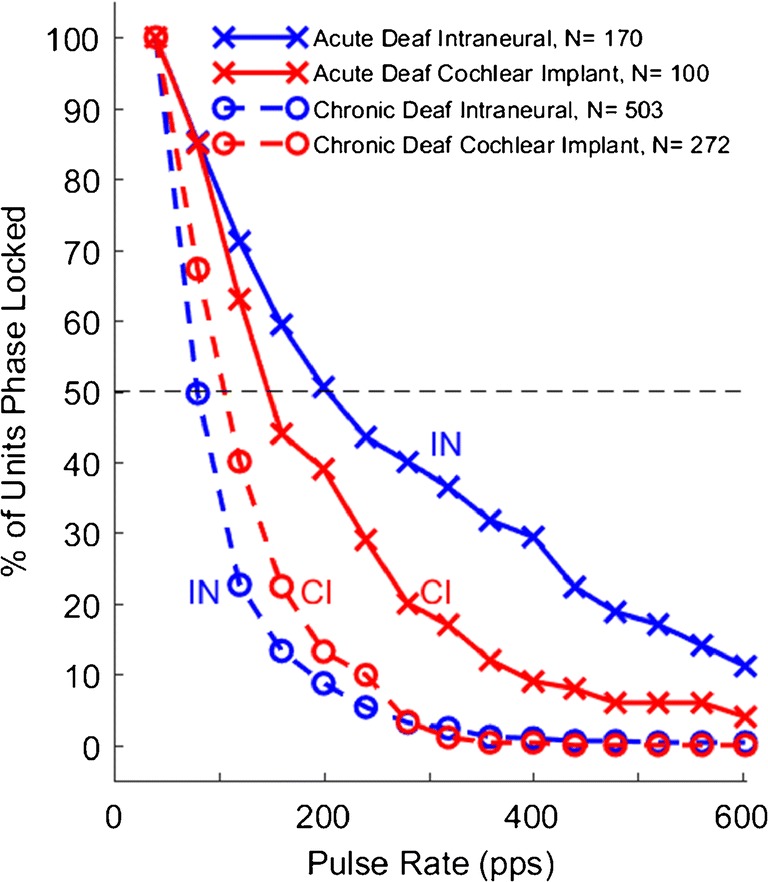
Percentage of ICC units showing significant phase locking to electrical pulse trains presented through cochlear implant (CI) or intraneural (IN) electrodes. Data are plotted as a function of electrical pulse rate in pulses per second (pps). Solid lines indicate data from five acutely deaf cats, re-plotted from Middlebrooks and Snyder (2010). Dashed lines indicate data from 12 chronically deaf cats from the present study. Stimulus levels were 0 to 4 dB above the threshold for each neuron’s response to a single electrical pulse according to the *d’* measure described in the “Methods” section with a criterion for threshold of *d’* = 1. Data are from every ICC unit that was above threshold and that synchronized to a minimum rate of 40 pps. One CI site and two to six IN sites were stimulated in each cat

Temporal acuity was evaluated by measuring for each ICC unit the *limiting rate*, which was the highest rate of electrical pulses to which the unit could phase lock significantly (as described in the “Methods” section). The distributions of limiting rates are shown in Fig. 7 as the percentage of ICC units that were phase locked as a function of electrical pulse rate. The data shown with solid lines are from the acutely deaf animals (re-plotted from Middlebrooks and Snyder 2010). As in the 2010 report, at any pulse rate ≥ 120 pps, a substantially higher percentage of units phase locked to IN stimulation (blue x’s; interpolated median limiting rate = 203 pps) than did to CI stimulation (red x’s; median = 147 pps) (Kolmogorov-Smirnov test, *k* = 0.20, *p* = 0.0088). The mean limiting rate was higher for IN than for CI stimulation in all five of the acutely deaf animals. The data shown with dashed lines are from the chronically deaf animals in the present study. For both CI and IN stimulation conditions, the chronically deaf animals exhibited a striking reduction in the percentage of phase-locked neurons at each pulse rate > 40 pps (CI acute versus CI chronic: *k* = 0.26, *p* = 0.000090; IN acute vs. chronic: *k =* 0.49*, p* < 1e^−6^). Interpolated median limiting rates were 79 pps for IN stimulation in chronic deaf animals and 105 pps for CI stimulation in those animals. In contrast to the acutely deaf animals, which showed greater phase locking to IN than to CI stimulation, the chronically deaf animals showed significantly greater phase locking to the CI stimulation (*k* = 0.18, *p* = 0.000030), although phase locking in the chronically deaf animals was poor for both stimulation conditions. The mean limiting rate was higher for CI than for IN stimulation in five of the six chronically deaf animals in which both IN and CI stimulation was tested.

**Fig. 8:**
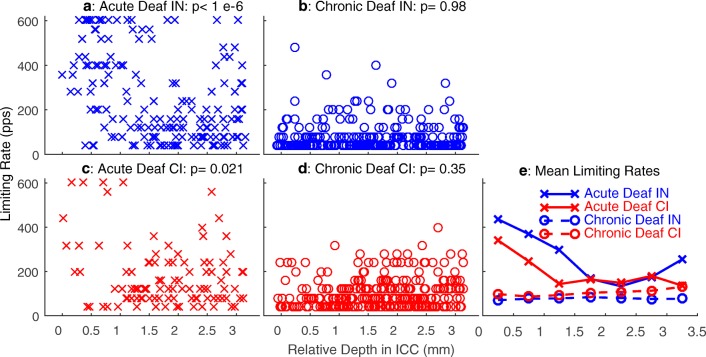
Limiting pulse rates for significant phase locking of ICC units as a function of recording depth in the ICC. Depths are given relative to the most superficial ICC recording site in each animal. Left panels (**a** and **c**) show data from the five acutely deaf cats re-plotted from Middlebrooks and Snyder 2010. Right panels (**b** and **d**) show data from the 12 chronically deaf cats in the present study. Data points are jittered in the horizontal dimension to improve visibility. **e** Mean values for each condition and each ICC depth, computed in 0.5-mm increments of depth

In our previous study (Middlebrooks and Snyder 2010), we found that limiting rates tended to vary with the characteristic frequencies (CFs) of ICC neurons, with the highest limiting rates measured from neurons having CFs lower than around 1.5 kHz. We could not test for a corresponding CF dependence in the present study, because the chronic deaf animals were deaf at the time of implantation of the ICC recording array and, therefore, the CFs could not be measured. Nevertheless, it is well established that CF tends to increase monotonically with depth in the ICC. For that reason, we examined the distribution of limiting rates as a function of ICC depth (Fig. 8). Here, the data from acutely deaf animals, previously plotted as a function of CF (Middlebrooks and Snyder 2010), are re-plotted as a function of ICC depth. Depths are given relative to the most superficial ICC recording site in each animal.

In the responses to IN stimulation in acutely deaf animals (Fig. 8a), one can see a population of units at depths superficial to ~ 1.5 mm in the ICC having limiting rates of 600 pps, which was the highest rate tested. In the acutely deaf animals, limiting rate varied systematically with ICC depth (one-way ANOVA, depths grouped in 0.5-mm increments: *p* < 1e^−6^, *F* = 10.6, *d.f.* = 6,163). There was a similar trend in the CI stimulation of the acutely deaf animals (Fig. 8c; *p* = 0.021, *F* = 2.6, *d.f.* = 6,93). The trend was weaker in the case of CI stimulation, primarily because of the small number of units that were activated at superficial depths by the CI stimulus. The chronically deaf animals showed no significant dependence of limiting rate on ICC depth for either IN (Fig. 8b; *p* = 0.98) or CI (Fig. 8d; *p* = 0.35) stimulation.

Figure 8e shows mean limiting rates as a function of ICC depth. Limiting rates in the chronically deaf animals clearly were degraded at all ICC recording depths, although the loss of acuity was most conspicuous in the superficial sites at which limiting rates were high in the acute animals. In our previous report of the acute deaf data (Middlebrooks and Snyder 2010), there was no significant difference in limiting rates between acute IN and acute CI after accounting for CF. Plotted here versus ICC depth, the acute IN and CI data show a significant difference in limiting rates, probably because the plot versus depth does not capture the under-sampling of low-CF neurons in the case of CI stimulation. The limiting rates from chronically deaf animals showed no dependence on recording depth but were significantly higher for CI than for IN stimulation (two-way ANOVA: IN versus CI, *p =* 0.000015, *F =* 19, d.f. = 1,543; depth: *p =* 0.66, *F =* 0.69). That limiting rates were higher for IN than for CI stimulation in acute deaf animals but lower for IN than CI in chronically deaf animals was unexpected and is discussed in the “Discussion” section.

**Fig. 9:**
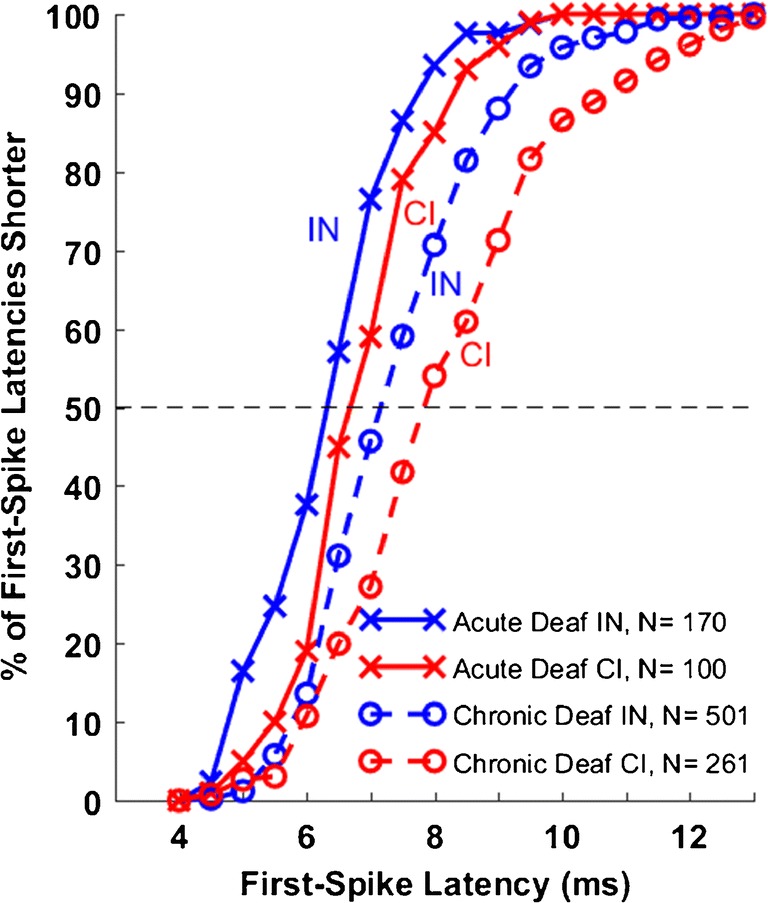
Distribution of first-spike latencies. The lines show, at each first-spike latency, the percentage of units having a latency equal to that value or shorter. Solid lines indicate data from the five acutely deaf cats, re-plotted from Middlebrooks and Snyder (2010). Other format details are as in Fig. 7

We hypothesized that the degraded temporal acuity evident in chronically deaf animals as reduced limiting rates might also manifest as elongated latencies from electrical cochlear stimulation to the first spikes of ICC neurons. Figure 9 shows the distributions of first-spike latencies for the various conditions; a symbol at a particular first-spike latency indicates the percentage of units having a latency at or shorter than that value. For both modes of cochlear stimulation, CI and IN, the distributions of latencies of ICC units in chronically deaf animals showed a substantial shift toward longer latencies relative to latencies in acutely deaf animals (Kolmogorov-Smirnov tests, CI: *p* < 1e^−6^, *k* = 0.378, IN: *p* < 1e^−6^*, k* = 0.0.31)*.* Latencies tended to be longer for CI than for IN stimulation both in the acutely deaf animals (as reported previously, Middlebrooks and Snyder 2010; *p* = 0.0024, *k* = 0.227) and chronically deaf animals (*p* < 1e^−6^, *k* = 0.0.21).

**Fig. 10:**
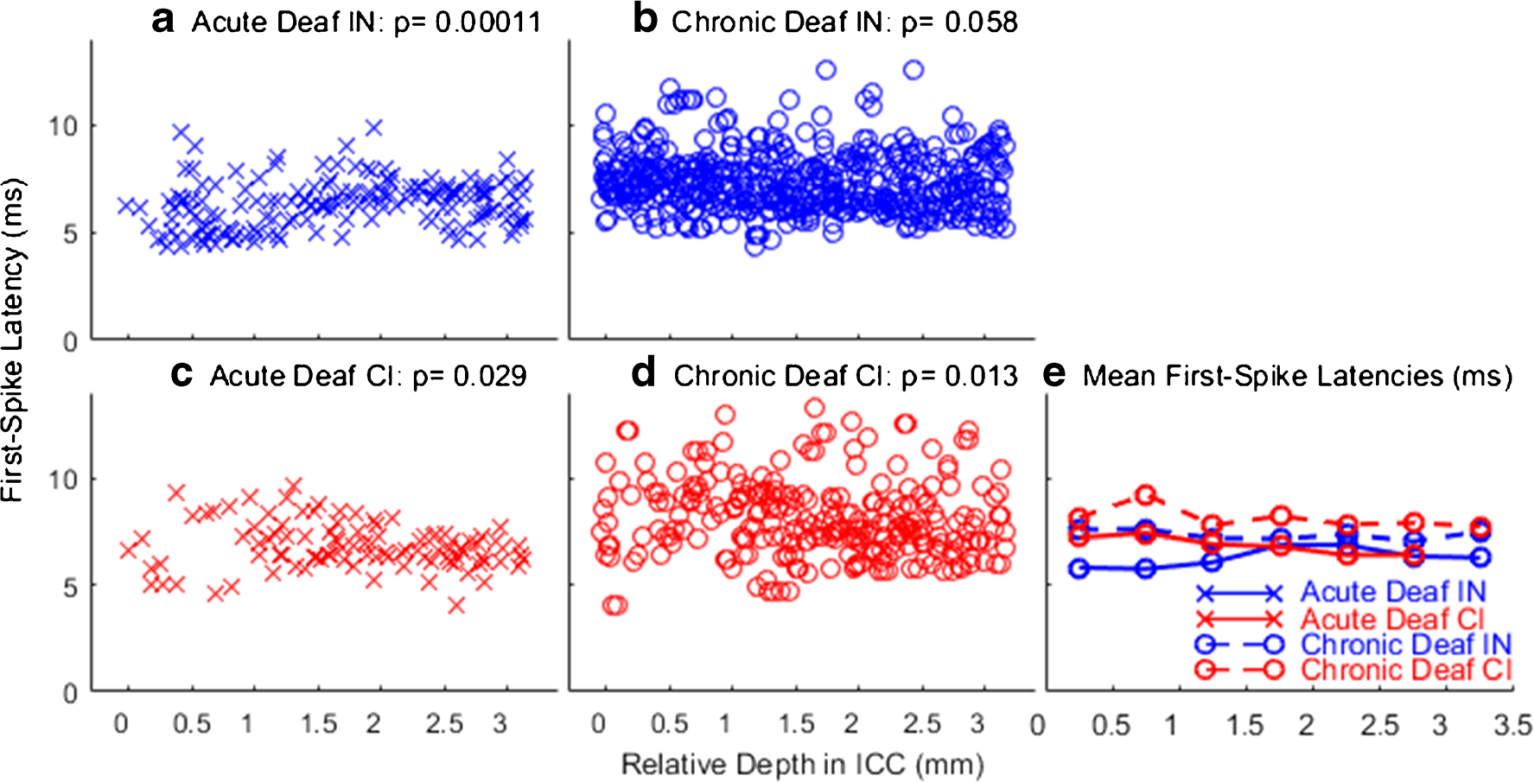
First-spike latencies as a function of recording depth in the ICC. Format details are as in Fig. 8

The distribution of first-spike latencies for the various stimulation and deafness conditions as a function of recording depth in the ICC is shown in Fig. 10. The responses to IN stimulation in the acutely deaf animals showed a significant variation with recording depth (Fig. 10a; *p* = 0.00011, *F* = 5.0, *d.f.* = 6,163), with the shortest latencies tending to be recorded at the most superficial depths. Such a trend was just barely significant after Bonferroni correction for CI stimulation in the acutely deaf animals (*p =* 0.029, *F* = 2.5, *d.f.* = 6,93). There was no significant dependence of first-spike latencies in the chronically deaf animals for IN stimulation (*p =* 0.58) but a slight trend for CI stimulation (*p =* 0.013, *F =* 2.8, *d.f. =* 6,254) stimulation. Mean values of first-spike latencies are plotted in Fig. 10e as a function of ICC recording depth. Among the acutely deaf animals, latencies were shorter for IN than for CI stimulation (two-way ANOVA: IN versus CI, *p =* 0.00023, *F =* 9.6, *d.f. =* 1,262; depth: *p =* 0.0011, *F =* 3.9, *d.f.* = 6,262). Post hoc analysis of those acute deaf data showed no significant difference between in latencies between IN and CI at specific ICC depths (*p >* 0.05 after Bonferroni correction). Among the chronically deaf animals, latencies were shorter for IN than for CI stimulation (two-way ANOVA: IN versus CI, *p <* 1e-6, *F =* 49.8, *d.f. =* 1,754; depth: *p =* 0.0022, *F =* 3.5, *d.f.* = 6,754). Post hoc analysis with Bonferroni correction showed shorter latencies (*p <* 0*.*05) for IN than for CI stimulations at all except the deepest ICC recording depths. That first-spike latencies were shorter for CI than for IN stimulation in the chronically deaf animals was unexpected given that, in those animals, limiting rates were higher for CI than for IN stimulation.

**Fig. 11:**
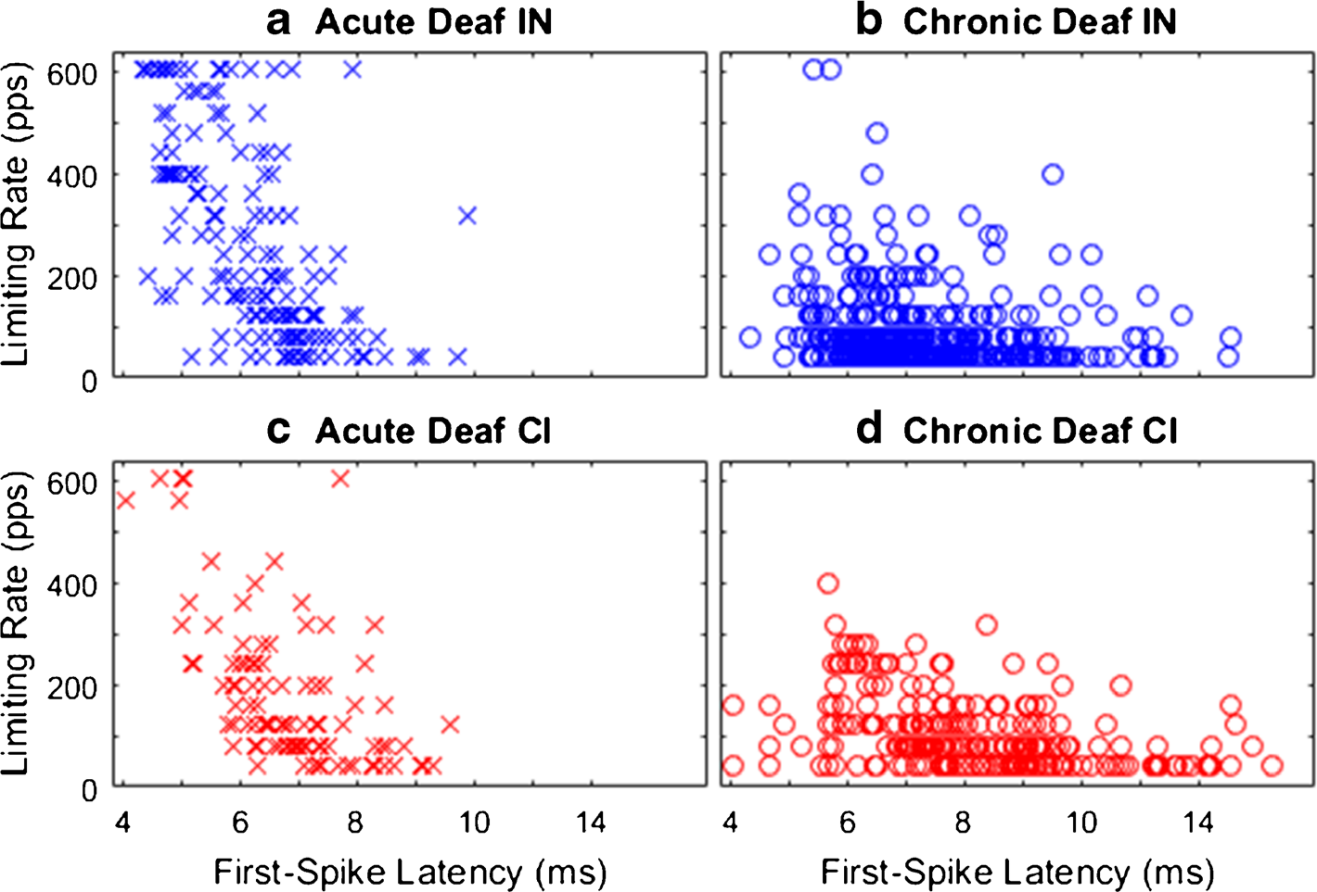
Relationship between limiting rate (vertical axis) and first-spike latency (horizontal axis)

The relationships between limiting rates and first-spike latencies are shown in Fig. 11. There was a general trend for higher limiting rates to be associated with shorter first-spike latencies (*p* < 1e-6, *F =* 5.3 to 20, depending on IN versus CI and acutely versus chronically deaf).

## Discussion

Cats that were deafened as adults and left unstimulated for around 6 months showed a substantial reduction in the numbers of myelinated fibers in their auditory nerves. Contrary to our expectation, that loss of fibers had essentially no effect on excitability of the auditory pathway monitored at the level of the ICC. This included no marked changes in tonotopic organization of the nerve, no change in thresholds for activation of ICC neurons, and no change in the dynamic ranges over which restricted tonotopic activation was maintained (i.e., in FSDRs). The most striking functional consequence of 6 months of deafness was a profound decrease in temporal acuity for both CI and IN stimulation. The degradation of temporal transmission will be discussed in regard to its implications for CI users and a possible role of chronic electrical stimulation in maintaining or restoring temporal acuity.

### Loss of Fibers during Chronic Deafness

It is well established that essentially all type I spiral ganglion cells have one peripheral process that innervates one and only one inner hair cell, and nearly all of these ganglion cells send a single myelinated axon in the auditory nerve to branch and synapse in the cochlear nucleus. For that reason, we anticipated that our counts of myelinated fiber counts in normal animals (Fig. 4) would approximate the estimated numbers of type I ganglion cells from normal-hearing cats in published reports (Howe 1934; Mair 1973; Gacek and Rasmussen 1961; Chen et al. 2010). These estimates of numbers of type I ganglion cells from 35 cat ears in published reports ranged from 44,298 to 57,541 (mean = 51,100); the 35 ears include 17 “functionally and histologically normal ears” in a study of hereditary deafness in white cats (Mair 1973). Our counts of myelinated auditory nerve fibers in 6 acutely deaf cochleas ranged from 54,423 to 76,649 (mean = 65,712), which is an average of 28.6 % more fibers in our control animals than ganglion cells in published reports. The most likely explanation for that apparent surplus of fibers is that our nerve sections were taken at a level intended to be sure to include all auditory nerve fibers. Those sections likely also included the inferior branch of the vestibular nerve. Despite the likely inclusion of vestibular fibers in both the deaf and control fiber counts, however, the ~ 30 % drop in the number of fibers observed in our chronically deaf animals falls within the range of percentage ganglion cell loss recorded in comparable studies of duration of deafness in cats (Leake et al. 2008).

### Degraded Temporal Acuity after Chronic Deafness

The most striking functional consequence of ~ 6 months of deafness is the marked loss of temporal acuity seen both as a reduction in limiting rates for phase locking to pulse trains and as an increase in first-spike latencies. We reported previously that the highest limiting rates and shortest first-spike latencies in acutely deafened cats are associated with low-CF pathways originating in the cochlear apex, primarily with CFs < 1.5 kHz (Middlebrooks and Snyder 2010). In that report, we argued that those low-frequency pathways most likely involve the large axosomatic end bulbs of Held, synapsing on the large spherical bushy cells (SBCs) of the anterior ventral cochlear nucleus (AVCN), projecting through the trapezoid body to the medial superior olive (MSO), and terminating on low-CF neurons in the ICC. The number of end bulbs on each SBC in cat is small, mostly two, ranging from one to four (Ryugo and Sento 1991). We observed a ~ 30 % loss of auditory nerve fibers after ~ 6 months of deafness. For the sake of discussion, we assume that the loss is uniform across cochlear turns, although we did not test that. Given the small number of auditory fibers terminating on each SBC, one would expect that an overall loss of 30 % of fibers could result in elimination of around half of the input to a sizeable percentage of SBCs.

The deafness-related loss of temporal acuity was most striking at superficial ICC depths corresponding to low CFs, primarily because those are the depths showing highest temporal acuity in the acutely deaf condition. Nevertheless, significant loss of acuity was observed at all ICC depths for both IN and CI stimulation. Deeper in the ICC (i.e., at higher CFs), the predominant contralateral excitatory input originates from type I multipolar (stellate) cells in the contralateral AVCN and posterior ventral cochlear nucleus (PVCN) and from the contralateral lateral superior olive (Cant and Benson 2003). The multipolar cells, having the characteristic “chopper” responses, receive axodendritic inputs from numerous auditory nerve fibers. For that reason, one would expect most or all multipolar cells to suffer some loss of input as a result of 30 % loss of auditory nerve fibers. In those cells, however, the loss of one or more nerve fibers presumably would be graded rather than as catastrophic as would be the case for SBCs.

In addition to the overt loss of auditory nerve fibers, the central auditory system likely undergoes changes as a result of months of inactivity in auditory afferents. Shrinkage of endbulbs of Held and of SBCs are particularly marked in congenitally deaf cats (Ryugo et al. 1997; O’Neil et al. 2010) but also can be observed in conditions of progressive hearing loss (in mice: Connelly et al. 2017). The cochlear nucleus exhibits an overall loss of volume following destruction of the cochlea in adult cats (Powell and Erulkar 1962) or ototoxic deafening of neonatal cats (Lustig et al. 1994; Hardie and Shepherd 1999). That loss of volume is due primarily to loss of neuropil and shrinkage of cells, not to loss of cochlear nucleus neurons. Deafness-related shrinkage of auditory brainstem neurons and neuropil has been reviewed by Shepherd and Hardie (2001).

Although we observed marked deafness-related losses of temporal acuity for both IN and CI stimulation, limiting rates in the chronically deaf animals were slightly but significantly higher for CI than for IN stimulation, the opposite of what we see in acutely deaf animals. One possible explanation for that result might be that, in a partially depopulated auditory nerve, the limited current spread from IN stimulation might be less effective in recruiting fibers for temporally synchronized activation than is the broader current spread from CI stimulation. Single electrical pulses from both IN and CI stimulation were adequate to produce near-normal thresholds and FSDRs, and first-spike latencies showed the same relation (i.e., IN shorter than CI) in chronically deaf animals as is seen in acute deafening. It might be the case, however, that somewhat larger activated nerve populations are needed to achieve high pulse-following rates than are needed for simple responses to single pulses or to the first pulse in a train.

Degraded temporal acuity in the pathway to the ICC has been evaluated in a series of studies involving neonatal deafening of cats and CI stimulation (Snyder et al. 1991, 1995; Vollmer et al. 1999, 2005); the upper limit of phase locking reported in those studies, Fmax, was the metric most comparable to the limiting rate of the present study. In those studies, the “normal” or “control” condition consisted of adult cats that were deafened 1.5–3.5 weeks prior to ICC recording; that contrasts with our acutely deaf adults that were deafened < 18 h prior to ICC recording. The mean and median Fmax in the previous studies were substantially lower than the median limiting rate for CI stimulation in the acutely deaf animals in the present study (first reported in Middlebrooks and Snyder 2010): mean Fmax was 102 pps (Vollmer et al. 1999) and median Fmax was 95 pps (Vollmer et al. 2005) compared to the median limiting rate of 147 pps for the CI array in the present study. Indeed, for CI stimulation, the median limiting rate in the chronically deaf condition in the present study, 105 pps, was closer to the published control values. One possible explanation for the ~ 50 % higher rate of phase locking for acute deafness in the present study is that the previous results from Snyder and Vollmer and colleagues might have reflected some loss of temporal acuity as soon as the 1.5 to 3.5 weeks between deafening and ICC recording. It is unlikely that there was substantial loss of nerve fibers over that time, but a few weeks of inactivity of afferents may well have led to some synaptic reorganization. Additional support for the notion that temporal acuity is greater within a few hours after deafening than after a few weeks of deafness is given by the study by Shepherd et al. (1999), who studied two adult cats within hours of deafening. That study used a much more conservative measure of phase locking than in the present study: a probability of one or more spikes in response to ≥ 90 % of electrical pulses. Nevertheless, the 75th percentile of maximum rates reported in that way was ~ 90 pps, suggesting that the median limiting rate or Fmax must have been well above 100 pps, comparable to the value that we obtained with a similar hours-long duration of deafness.

Hancock et al. (2012, 2013) have reported an influence of the age of deafening on temporal acuity using intrascalar CIs. They studied congenitally deaf white cat, adult cats deafened 1–2 week prior to study, and adult cats deafened ~ 6 months prior to study. A cluster analysis was performed based on plots of sustained neural firing rate versus electrical pulse rate. The percentage of neurons that responded to pulse rates > 20 pps was highest in the 1–2-week deaf group, lower for 6-month deaf, and lowest for congenitally deaf (Hancock et al. 2012). Using a measure of limiting rates, however, the lowest mean limiting rates were found among the congenitally deaf group, but there was no significant difference between 1–2-week and 6-month deaf groups. The latter observation accords with the notion that a considerable amount of temporal acuity can be lost within ~1–2 weeks following deafening of an adult cat.

The degradation of temporal acuity observed in the present study in the condition of cats deafened as adults and left unstimulated for ~ 6 months was so severe that one might wonder how it is that cochlear implants are as successful as they are in post-lingually deaf patients that are implanted as adults. The answer might be that temporal acuity of the auditory pathway is maintained or improved by electrical stimulation with a cochlear implant. The main objective of the studies by Snyder and Vollmer and colleagues (Snyder et al. 1991, 1995; Vollmer et al. 1999, 2005) was to evaluate the degree to which chronic electrical stimulation might prevent or reverse a loss of temporal acuity in cats deafened as neonates. Animals that were deafened as neonates, left unstimulated through development, and studied as adults showed a dramatic loss of temporal acuity compared to the controls. Median Fmax for deafened neonates, unstimulated and then studied as adults, was 70 pps compared to 95 pps for 1.5–3.5 weeks of deafness as an adult (Vollmer et al. 2005) and compared to the median limiting rate of 105 pps for the chronically deaf cats in the present study. One assumes that the greater impact of deafening on neonates is a consequence of the absence of afferent activity during months of development. Indeed, restoration of patterned nerve activity with a cochlear implant in neonatally deafened cats improved their temporal acuity as adults. That is, chronic stimulation at a low pulse rate (≤ 80 pps) maintained the median Fmax at 109 pps, comparable to controls, and chronic stimulation with a higher-frequency modulated pulse train actually elevated the median Fmax to 134 pps (Vollmer et al. 1999), which is close to our acutely deaf adult limiting rates. It might be that degradation of temporal acuity in human implant users is avoided by the stimulation that they receive through everyday use of their cochlear implants. In future studies, we hope to evaluate effects of chronic electrical stimulation in animals that were deafened as adults as a model of benefits of cochlear implantation in post-lingually deaf patients.
